# Evaluation and improvement the safety of total marrow irradiation with helical tomotherapy using repeat failure mode and effects analysis

**DOI:** 10.1186/s13014-019-1433-7

**Published:** 2019-12-27

**Authors:** Jiuling Shen, Xiaoyong Wang, Di Deng, Jian Gong, Kang Tan, Hongli Zhao, Zhirong Bao, Jinping Xiao, An Liu, Yunfeng Zhou, Hui Liu, Conghua Xie

**Affiliations:** 1 Department of Radiation and Medical Oncology, Zhongnan Hospital of Wuhan University, 169 Donghu Road, Wuhan, Hubei 430070 People’s Republic of China; 2 Hubei Radiotherapy Quality Control Center, Wuhan University, Wuhan, Hubei China; 3 Divisions of Radiation Oncology, City of Hope National Medical Center, Duarte, CA USA

**Keywords:** Failure mode and effects analysis, Total marrow irradiation, Helical tomotherapy, Process quality assurance

## Abstract

**Background & purpose:**

Helical tomotherapy has been applied to total marrow irradiation (HT-TMI). Our objective was to apply failure mode and effects analysis (FMEA) two times separated by 1 year to evaluate and improve the safety of HT-TMI.

**Materials and methods:**

A multidisciplinary team was created. FMEA consists of 4 main steps: (1) Creation of a process map; (2) Identification of all potential failure mode (FM) in the process; (3) Evaluation of the occurrence (O), detectability (D) and severity of impact (S) of each FM according to a scoring criteria (1–10), with the subsequent calculation of the risk priority number (RPN=O*D*S) and (4) Identification of the feasible and effective quality control (QC) methods for the highest risks. A second FMEA was performed for the high-risk FMs based on the same risk analysis team in 1 year later.

**Results:**

A total of 39 subprocesses and 122 FMs were derived. First time RPN ranged from 3 to 264.3. Twenty-five FMs were defined as being high-risk, with the top 5 FMs (first RPN/ second RPN): (1) treatment couch movement failure (264.3/102.8); (2) section plan dose junction error in delivery (236.7/110.4); (3) setup check by megavoltage computed tomography (MVCT) failure (216.8/94.6); (4) patient immobilization error (212.5/90.2) and (5) treatment interruption (204.8/134.2). A total of 20 staff members participated in the study. The second RPN value of the top 5 high-risk FMs were all decreased.

**Conclusion:**

QC interventions were implemented based on the FMEA results. HT-TMI specific treatment couch tests; the arms immobilization methods and strategy of section plan dose junction in delivery were proved to be effective in the improvement of the safety.

## Introduction

Total marrow irradiation (TMI) has been an important part of conditioning regimens for patients undergoing hematopoietic cell transplantation [1–3]. TMI treated with helical tomotherapy (HT-TMI) have been proved to be a clinically feasible way with a uniform and accurate dose distribution to the target and a significant reduction in side effects [4–8]. The long irradiation target volume makes the process much more complex compared to the conventional radiotherapy for tumors. Any potential errors can lead to serious treatment failure and tighter tolerances should be placed on QC methods. In addition to implement tighter tolerances of the routine daily and monthly equipment-related QC, the importance of human factors should not be ignored [9–11]. Although, many centers have published their application of HT-TMI, Unfortunately, no one has reported the safety and QC of HT-TMI process, and the existing potential errors haven’t been figured out and analyzed, which could be very dangerous and imperative.

FMEA as a prospective risk method has been applied to analyze radiotherapy processes by Scorsetti [12] first, and proved to be an effective method for quality improvement [13–21]. FMEA was organised into four main steps: (1) process analysis, (2) identification of the FMs, (3) risk analysis, (4) identification of corrective actions. In our study, the score team consists of a manager of the Medical Quality Control Committee of our department, a chief physicist and a chief therapist and the regular physician, physicist and therapist who have obtained a qualification certificate and participated in the daily clinical treatment process with a range of number of years experience.

## Materials and methods

### HT-TMI technique

**Fig. 1:**
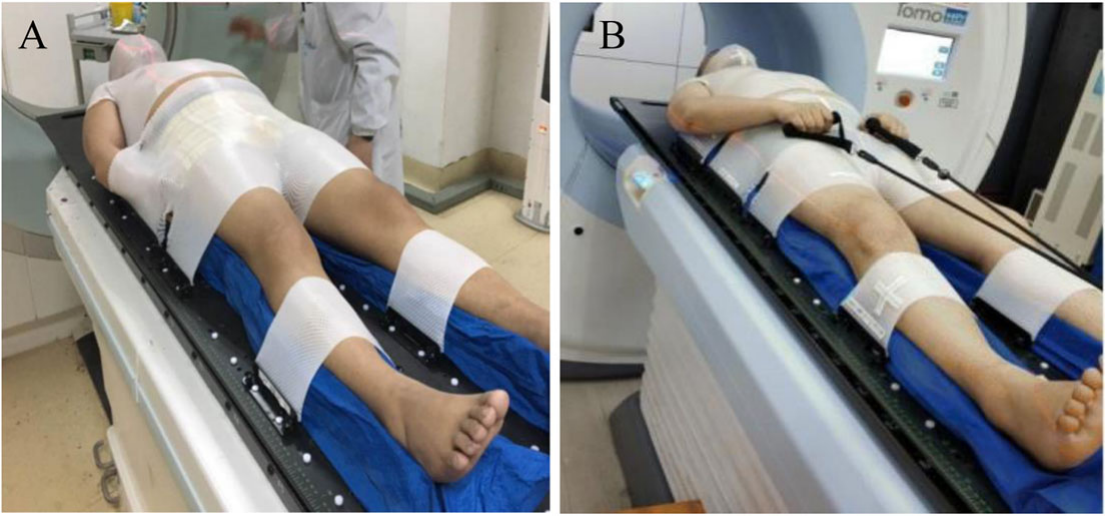
Example of the use of the 3 thermoplastic masks for patient immobilization. a first immobilization system and b new immobilization system

The HT-TMI program at our institution started in 2016. More than 80 patients have been treated. The process of HT-TMI is completed in 1 week, with a total prescription of 8 Gy delivered by 2 fractions (4 Gy per fraction) separated by ≥6 h in 1 day. As is shown in Fig. 1a, all patients were immobilized using a home-made dedicated immobilization system. CT scans, treatment planning and delivery details can be found in our previous study [22].

### FMEA process

A multidisciplinary risk analysis team was established consisting of two nurses, five medical physicists, four physician and nine therapists (details shown in Table 1). The team was led by a manager of the Medical Quality Control Committee of our department, and all members were involved in the scoring process. The FMEA was performed in several steps (in order):

1. Every step in the process was identified by each member with regard to his or her own area of expertise. Because of the level of details of each step was found to be different among members, discussion was carried until the team reached a consensus on the steps within each subprocess. The goal was to ensure each step was clear and manageable. After that, all steps were wrote down and laid out in the order to build the process tree.
2. Identification of failure modes (FM) for each step as many as the team could imagine. The possible causes and clinical situations where the failure could occur as well as S were listed with their collective experience.
3. Each FM was assigned three scores (O, D and S) of 1 to 10 with a consensus 10-point scale (shown in Table 2) based on their individual experiences. RPN value was derived by multiplying the three individual scores with each other. The averaged O, D, RPN value and maximum S score of all members was defined as the final score. FMs were ranked by RPNs from highest to lowest, the top 20% were defined as “high-risk”.
4. Fault tree analysis was performed to visualize the locations and propagation of FMs through the process. Appropriate quality management (QM) program were implemented to mitigate the most important risks which were identified in the previous analyses.
5. Second FMEA was performed for the high-risk FMs follow the steps 3 above based on the same risk analysis team in 1 year later to verify the effectiveness of the QM program.

**Table 1:** Details in the composition of the scoring team, the total number of scorers and the number of scorers with different working years in each sub-process

Sub_process	Members	Number of scorers with different years of experience			Total
		1–3 years	3–5 years	> 5 years	
Registration	Nurse	0	1	1	2
Immobilization /CT simulation Image transmission	Therapist	1	1	2	4
Contouring /Prescription Plan approval	Physician	2	1	1	4
Treatment planning/evaluation Plan preparation	Physicist	2	2	1	5
Treatment preparation/delivery	Therapist	2	2	1	5

**Table 2:** Discription of 10-point scales used to derive values of probability, severity, and detectability for each failure mode

Rank	Occurence(O)	Severity(S)	Detectability(D)
1	Very low; less than 2%	No effect	Very easy to detect; alomost never miss
2		Inconvenience; discomfort or distress	
3	Low; approximately 5%		Easy to detect, double check should be performed
4		Minor dosimetric error; approximately 5%	
5	Moderate	Limited toxicity or underdose; 10% dose difference with insufficient accutacy	Moderate, a “lucky catch”
6	Medium; occasional failure		Very difficult to detect a subtle error in mechanical position or highly unusual situation, e.g., treatment table auto setup error
7		Serious toxicity or underdose; 20% dose difference, wrong site irradiation	
8	High; repeated failures		
9		Very serious toxicity; patient injury or death	Almost impossible to detect
10	Inevitable		

## Results

**Fig. 2:**
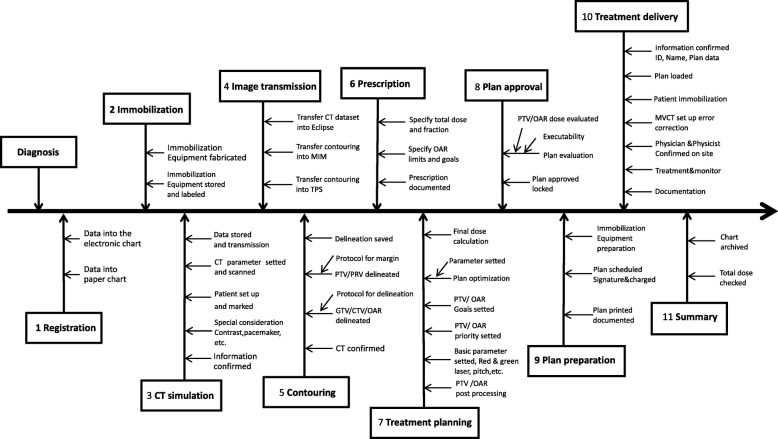
Process map of total marrow irradiation with helical tomotherapy

HT-TMI process map is shown in Fig. 2. Thirty-nine subprocess and 122 FMs were identified with a total of 20 members participating.

In the first time FMEA, RPN values range from 3 to 264.3 with a median value 30 and average value 42, the average value of O, D and S were 2.0, 2.3 and 4.8, respectively. A total of 25 high-risk FMs were found, and top 5 FMs (first RPN/ second RPN) are (details shown in Table 3): (1) treatment couch movement failure (264.3/102.8); (2) section plan dose junction error in delivery (236.7/110.4); (3) setup check by MVCT failure (216.8/94.6); (4) patient immobilization error (212.5/90.2) and (5) treatment interruption (204.8/134.2). The RPN value of the top 5 high-risk FMs found in the first FMEA were all decreased and no longer high-risk FMs in the second FMEA after adaption of the QM program. A total of 20 new high-risk FMs were found and appropriate QM program were implemented.

**Table 3:** Top 5 failure mode, occurrence (O), detectability (D) and risk priority number (RPN), subscripts 1 and 2 indicate the first time and second time scores respectively

NO.	Sub_process	FM	O_1_/O_2_	D_1_/D_2_	S_1_/S_2_	RPN_1_/RPN_2_
1	Treatment delivery	Treatment couch movement failure	5.2/4.8	6.5/3.2	8/8	264.3/102.8
2	Treatment delivery	Section plan dose junction error in delivery	4.8/4.5	5.6/3.1	9/9	236.7/110.4
3	Treatment delivery	Setup check by MVCT failure	6.5/3.3	4.5/3.5	7/7	216.8/94.6
4	Immobilization	Patient immobilization error	7.5/3.2	3.6/3.6	8/8	212.5/90.2
5	Treatment delivery	Treatment interruption	8.5/5.5	3.2/3.2	7/7	204.8/134.2

### Treatment couch movement failure

The delivery of a HT-TMI plan requires the accuracy of table movement with approximately 160 cm in length. Since the helical tomotherapy system was commissioned, the couch tests were performed on a monthly basis followed by the recommendation of AAPM TG148 [23]. During the delivery of HT-TMI, the subtle couch movement error was very difficult to detect by the traditional test with only a movement distance of 20 cm, so the team gave a D score 5.2 and the highest RPN value 264.3. After group discussion, the test of the couch was redesigned, the measuring distance is extended from the previous 20 to 160 cm, the tolerance between physical distances traveled and the digital readout at different couch heights was set to be within 3 mm, the leveling of the stationary couch should be less than 0.5° and the lateral couch position should deviate by less than 3 mm. In addition, the test frequency from the previous monthly becomes the measurement before each treatment.

### Section plan dose junction error in delivery

Dose homogeneity at the junction region from the overlap of the two plans was considered in the treatment planning stage [22]. In the delivery stage, we have performed three MVCT scans in Plan-upper and one for Plan-lower [22], but no scans covered the dose junction region, after couch shifts of the fourth MVCT scans, the three consecutive slices (used for section plan dose junction) from the Plan-upper and Plan-lower may mismatch. Resulting in hot-spots and cold-spots near the junction region. After group discussion, we demand that an additional MVCT scan covering the dose junction region must be performed in the delivery of Plan-upper, and the longitudinal distance of the fiducial markers between planning CT and MVCT is measured. To complement the dose distributions of junction region from Plan-upper, in the Plan-lower delivery stage, the shifts in SI direction were set to the opposite value (Plan-lower is delivered in FFS) of the measured distance above to match the setup error in SI direction from Plan-upper.

### Setup check by MVCT failure

In HT-TMI, three MVCT scans and segmented treatments for the Plan-upper were implemented to guarantee the treatment accuracy. However, considering factors such as the dose uniformity of the interruption position, the MVCT scan dose and efficiency of delivery, it was sometimes necessary to follow the principle of reducing the segmented treatments as much as possible when the patient was well immobilized and setup was precise. Sometimes the whole body setup check (assessment of the whole body setup deviation) by MVCT in the early setup stage was not performed by therapists who expected that the later three MVCT scans could eliminate all the setup errors in LR, SI and AP directions, as well as rotation around the SI axis. But in fact, if the patient was not setup perfectly, especially rotation around the AP axis, often more than three scans thus two interruptions (i.e., over-scan and over-interruption) must be performed to reduce the setup errors in the treatment stage. That resulted in an increased scanning dose, a risk of underdose or overdose near the interruption position and a prolonged treatment time. So the risk team gave the setup check by MVCT failure a RPN value of 216.8. With these concerns, we demand that therapists must perform three short scans (scan 1, orbits to the first cervical vertebra (C1); scan 2, seventh thoracic vertebra (T7 to T10); scan 3, fifth lumbar vertebra (L5) to sacrum.) to check the whole body setup deviation by MVCT after setup. If the difference between the three scans is more than 5 mm in any of the three translation directions and 1°in roll, the patient needs to be re-setup again.

### Patient immobilization error

Before first FMEA, shoulders and arms of the patients were immobilized close to both sides of the body and all covered in the masks. During treatment, if the whole body’s transverse diameter exceeded 40 cm, part of the shoulders and arms could not be fully captured by MVCT, because of the max field-of-view of HT-MVCT is only 40 cm in diameter. Therefore, the PTVs in the shoulders and arms were outside of the field-view where could not be checked precisely. After first FMEA, we developed a new method of immobilizing the shoulders and arms. Briefly, shoulders of the patients were immobilized close to both sides of the body and covered by the first mask, arms were positioned close to the thorax outside of the second mask; while hands were positioned on the groins with fingers grasping the rope to ensure good reproducibility (Fig. 1b). In this way, most of the PTVs in shoulders and arms could be seen in the MVCT images with only a little lateral soft tissue in the shoulders outside of the MVCT field view. Therefore, this new method was applied to improve the accuracy of the image fusion and treatment.

### Treatment interruption

Each TMI treatment takes nearly 2.5 h, which is a huge challenge for patients. During the course of treatment, if any urgent symptoms occur, such as vomiting, fever, convulsions, treatment must be stopped immediately. Because of large dose fraction (4–5 Gy/fraction) for the total body irradiation in our study, nearly half of patients develop acute radioactive reaction, especially in the second fraction. With these reasons, the risk team gave the interruption failure an O score of 8.5 and RPN value of 204.8. After group discussion, we recommended that sedative, tropisetron and antipyretic should be used more often to reduce adverse symptoms, before first FMEA, sedative was only used for little child, who couldn’t stay still during treatment.

### Second FMEA results

Second FMEA was performed for the high-risk FMs based on the same risk analysis team in 1 year later. Thirty-two patients were treated with HT-TMI in this year. The second RPN value of the top 5 high-risk FMs were all decreased (details shown in Table 3). For the treatment couch movement failure, we tested the accuracy of the treatment couch before every treatment and found four times has exceeded the tolerance, treatment started until the treatment couch was repaired by the engineer. D score of the FM has dropped to 3.2 and RPN of 102.8. In the section plan dose junction error, an additional MVCT scan covering the dose junction region was performed to complement the the setup error in SI direction from Plan-upper and Plan-lower and found three times the shifts in SI direction has exceeded 10 mm in this year, if without complement, the dose of the junction region could be seriously underdose or overdose. So the risk analysis team gave the FM with an D score of 3.1 and RPN of 110.4. For the setup check by MVCT failure, standard three short scans rules was applied and six patients have been re-setup again, both O, D and RPN score have dropped to 3.3, 3.5 and 94.6. For the patient immobilization error, after the new method of immobilizing the shoulders and arms was used, all the patients’ PTVs in shoulders and arms could be seen in the MVCT images. So the O score was assigned with 3.2 and the final RPN was 90.2. The probability of treatment interruption was also decreased after sedative, tropisetron and antipyretic was used more often with an O score of 5.5 and RPN of 134.2. The top 5 high-risk FMs found in the first FMEA were all decreased and no longer high-risk FMs in the second FMEA after adaption of the QM program. A total of 20 new high-risk FMs were found and appropriate QM program were implemented based on the fault tree analysis.

## Discussion

This paper used FMEA approach two times separated by 1 year to systematically uncover and analyze major FMs in a high risk clinical procedure - total marrow irradiation delivered with HT and propose feasible mitigations for the highest risk in the process.

In our study, the multidisciplinary team has a total of 20 members and not only consisted of a manager of the Medical Quality Control Committee of our department, a chief physicist and a chief therapist but also the regular physicians, physicists and therapists who participated in the daily clinical treatment process with varies number of years of working experience, which could make the FMs more comprehensive and close to the clinical procedure [24].

HT-TMI is a time consuming procedure with high precision, which puts high pressure on the machine and patient [4, 5]. The treatment couch movement failure was assigned with the highest RPN, although quality assurance methods of the couch have been supported by some literature [23], the distance of couch movement test was only 20 cm, which can not meet the demand of HT-TMI. For patient, the long treatment time was also a huge challenge, the treatment interruption failure was the fifth top FM, although Taiki Magome et al. [25] noted the fast megavoltage computed tomography approach can be used to shorten the scanning and image registration time, the treatment time can no longer be compressed due to the delivery mode of tomotherapy.

TMI is typically treated in 2 parts: an upper (body) and lower (legs) section, dose homogeneity at the junction region should be considered from the overlap of the two plans. Timothy E. Schultheiss et al. [4] has reported the dose junction method between tomotherapy and conventional linac, but the dose of the overlap region was 50% isodoses from the 2 treatment modalities. Mancosu et al. [26] has demonstrated the approach to create field junction from two volumetric modulated arc therapy plans with different patient orientation and obtained optimal target coverage of the treatment of TMI-TMLI. In our study, to complement the dose distributions of junction region from Plan-upper, in the Plan-lower delivery stage, the shifts in SI direction were set to match the setup error in SI direction from Plan-upper, which could be more accurate, but demand more steps and care. In terms of an FMEA approach, the method proposed by Mancosu et al. [26] may have the lower O and S scores of the dose junction error, because differences < 1% were found for mean doses to PTV and surrounding healthy tissue in the three directions regarding the 5 mm shifts. In our study, if the three consecutive slices from the Plan-upper and Plan-lower mismatched for 5 mm, which could generate 110% hot-spots near the junction region.

Many centers use different immobilization and pre-treatment position verification techniques. Two methods of pretreatment imaging were used: whole body MVCT imaging and partial body MVCT imaging [27]. But in fact, if the patient was not setup perfectly, often one whole body MVCT imaging or three scans can not reduce the setup errors well. In our study, three short scans were used to assess the whole body alignment in the early setup stage, if necessary, the patient should be re-setup. The new and feasible method of immobilizing the shoulders and arms was applied to improve the accuracy of the image fusion and treatment, which could effectively minimize the shoulder misalignment reported by Zuro et al. [27].

In our study, we creatively performed FMEA two times separated by one year to verify the effectiveness of the QC program and found that the RPN value of the high-risk FM were all dropped. The QC program introduced by the first time FMEA did help us solve some of the high-risk FMs and the second FMEA has found some new high-risk FMs. Two or more times FMEA can help us to build the PDCA cycle (plan do check act cycle) and improve the QM program continuously.

HT-TMI is an uncommon technique for many radiotherapy centers, but the complexity and potential errors of it should not be ignored. After performing the FMEA, we have uncovered several high-risk steps in our process and redesigned the QC method, although the results may not apply to other centers who have conducted TMI in other way or even in the same way, the findings of the section plan dose junction technique and immobilization methods of shoulders and arms could be useful for centers treated TMI with HT.

Our study had several limitations. One was that the study was a single group analyzing an uncommon procedure. The results might not apply to others. To minimize this shortcoming, we enlarged the group numbers to a total of 20 people and cooperated with other centers that have the similar HT-TMI process. Second was that some solutions to particular failure modes added additional tasks to the process. For example, additional MVCT scan could lead new errors. Therefore, to properly use the results of FMEA, we should combine with the fault tree analysis to make the errors more visible to implement efficient QC method and try to not modify the process too much.

## Conclusion

It can be concluded that QC interventions were implemented based on the FMEA results. The HT-TMI specific treatment couch tests; arms immobilization methods and strategy of section plan dose junction could be used to improve the safety.

## Data Availability

Please contact author for data requests.

## Abbreviations

AP: anterior and posterior
C1: first cervical vertebra
CT: computed tomography
D: detectability
FFS: foot first superior
FM: failure mode
FMEA: failure mode and effects analysis
HT-MVCT: megavoltage computed tomography of helical tomotherapy
HT-TMI: total marrow irradiation treated with helical tomotherapy
L5: the fifth lumbar vertebra
LR: left and right
MVCT: megavoltage computed tomography
O: occurrence
PDCA: plan do check act
QC: quality control
QM: quality management
RPN: risk priority number
S: severity of impact
SI: superior and inferior
T10: the tenth thoracic vertebra
T7: the seventh thoracic vertebra
TMI: total marrow irradiation
